# Successful control of a large intrahepatic cholangiocarcinoma treated by transarterial chemo-embolization; a single case report

**DOI:** 10.1259/bjrcr.20210186

**Published:** 2022-02-11

**Authors:** Atsushi Hori, Hiroshi Toei, Tatsuya Nakamura, Kazuhiro Makitani, Shinichi Hori

**Affiliations:** 1 Institute for Image Guided Therapy, Department of Interventional Radiology, Izumisano, Japan; 2 Department of Radiology, Juntendo University, Tokyo, Japan; 3 Department of Radiology, Wakayama Medical University, Wakayama, Japan

## Abstract

Intrahepatic cholangiocarcinoma is hardly diagnosed in early stages as the symptoms are non-specific. Due to an advanced stages at the time of first diagnosis, the therapeutic options for patients with unresectable cholangiocarcinoma are mostly limited to systemic chemotherapy or radiotherapy, but good local control or preferable prognostic effects are hardly obtained. The transarterial chemoembolization had not been a standard of care because of hepatic functional damages caused by lipiodol and gelatin sponge. A newly developed spherical embolic material causes limited hepatic damages might be an option for these patients. It makes it possible to repeat the procedure in a short period. Eventually, better prognosis can be expected using a spherical embolic material. We report a case of a 15 cm locally advanced intrahepatic cholangiocarcinoma treated by chemoembolization using a drug-eluting spherical embolic material and achieved good local tumor control without liver damage. The patient survived longer than 4 years without additional or concomitant treatments.

## Clinical presentation

**Figure 1.**
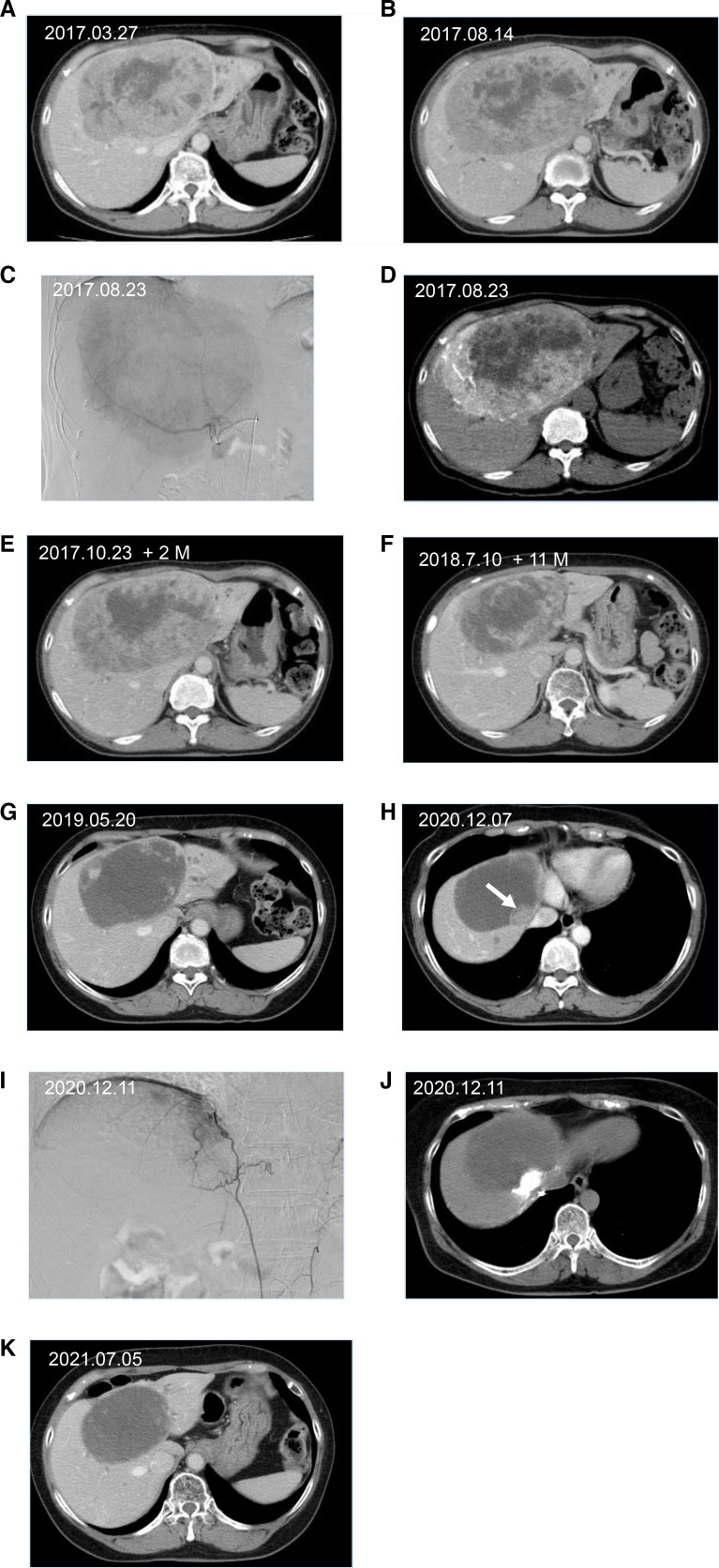
CECT and DSA images of the patient’s cholangiocarcinoma (IHCC). (a) Enhanced CT CECT 1 month after introduction of systemic GC chemotherapy in March 2017. (b) Enhanced CT before the first treatment 6 months after introduction of systemic chemotherapy. (c) CECT before the first TACE treatment August 2017 and a 15cm IHCC identified. (d) DSA images taken in the first procedure with intra-arterial chemoinfusion in proper hepatic artery and a vague vasculature in the liver was found. (e) CECT image 2-months after the first TACE with stable disease. (f) CECT images 11-months after the first TACE and seven sessions of TACE were done. Significant decrease in tumor size was demonstrated. (g) CECT image 22-months post initial TACE and 11 sessions of TACE were done. Tumor size was measured to be 11 cm and partial response was determined. (h) In 39 months, local recurrence was noted (white arrow) in the inner margin of necrotic tumor. (i) The right inferior phrenic arteriography showed feeding artery towards the tumor and the 13th TACE was performed. (j) Angio CT showed tumor enhancement with contrast injection in the right inferior phrenic artery. (k) CECT image showed a complete necrosis 4-years after the first TACE with no intrahepatic spread. Tumor size was measured to be 10cm. CECT, contrast enhanced CT; DSA, digital subtraction angiography; IHCC, intrahepatic cholangiocarcinoma; TACE, transarterial chemoembolization.

A 74-year-old female with good past health was admitted in August 2016 for Imaging investigation for an elevation of ALP and transaminase. CT of abdomen revealed a large tumor with 12 cm in diameter locating in the liver hilum with intrahepatic metastases. Patient was diagnosed as intrahepatic cholangiocarcinoma (IHCC) by percutaneous biopsy, and was treated with five cycles systemic chemotherapy using gemcitabine and cisplatin from February 2017 (Figure 1a). Treatment was terminated because of tumor progression and leukocytopenia. The patient was referred to our institution for (Image Guided Therapy) consultation in August 2017.

## Imaging findings

A contrast-enhanced CT (CECT) was performed. A large tumor with 15 cm in diameter was demarcated in the liver hilum accompanied with three intrahepatic metastases which were less than 20 mm in diameter (Figure 1b). Distant metastases or lymph node metastases were not detected. The tumor stage was T3N0M0.

## Treatment and outcome

She presented without specific symptoms with ECOG 0 and physical examination was unremarkable. Slight elevations of CA19-9, ALP, AST, ALP, LDH were observed (Table 1). Based on her previous treatments, we believed that systemic chemotherapy was no longer effective and introduced hepatic arterial chemoembolization therapy using drug-eluting microspheres. She then underwent celiac, superior mesenteric and hepatic angiography. The first TACE was carried out on August 23, 2017 with chemoinfusion using 30 mg Doxorubicin, 500 mg 5-FU and 6 mg Mitomycin. Each drug was diluted into 20 ml of saline. Immediately after infusion, embolization was followed using 22 mg of Doxorubicin-loaded HepaSphere (50–100 μm) from the proper hepatic artery (Figure 1c,d). No additional treatments were necessary to control adverse events after the procedure. She was discharged from hospital uneventfully 2 days after treatment.

**Table 1.** Blood tests

	Before initial TACE August 14, 2017	1 month after TACE September 15, 2017	After 4 years July 5, 2021
Albumin (3.7–5.3 g dl^−1^)	4.5	4.4	4.1
Total bilirubin (0.2–1.2 mg dl^−1^)	0.6	0.5	0.6
ALP (100–350 U l^−1^)	428	449	[td]
AST (10–40 U l^−1^)	62	49	22
ALT (6–40 U l^−1^)	85	48	16
LDH (120–240 U l^−1^)	273	225	229
WBC (35-91)	45	34	37
Hemoglobin (11.3–15.2 g dl^−1^)	11.7	11.5	12.5
Platelet (14.0-36.0 X10^4)^	17.3	18.5	19.5
CA19-9 (0–37 U ml^−1^)	42	40	8
CEA (0.0–5.0 ng ml^−1^)	3.0	2.2	1.2

ALP, alkaline phosphatase; ALT, alanine aminotransferase; AST, aspartate aminotransferase; CEA, carcinoembryonic antigen; LDH, lactate dehydrogenase; TACE, transarterial chemoembolization ; WBC, white blood cell.

The patient was followed up 1 month post-treatment and confirmed that the tumor did not grow in size and the contrast enhancement decreased in the center of tumor (Figure 1e). The same treatment was uneventfully repeated 10 times every 1–3 months with significant tumor necrosis and shrinkage for 22 months after the initial TACE. The patient showed no adverse event along with the treatment and no vessel damage observed (Figure 1g). In order to achieve complete response, in June 2019, the 12th treatment was performed with intrahepatic artery infusion using 10 mg Doxorubicin, 250 mg 5-FU, 10 mg CDDP and 100 mg Bevacizumab and embolized with doxorubicin loaded HepaSphere (50–100 μm). The patient has been in good condition and followed with imaging at 3-month intervals.

Local recurrence was observed In March 2021 and the 13th treatment was performed with chemo-infusion in the proper hepatic artery and right inferior phrenic artery using 20 mg Doxorubicin, 250 mg 5-FU, 10 mg CDDP, and 100 mg Bevacizumab and embolized with 3.0 mg of doxorubicin loaded HepaSphere (Figure 1i,j). Follow-up CT scan demonstrated a complete necrosis of main tumor without extrahepatic spread (Figure 1k). Small intrahepatic metastases which were detected at the first treatment had been stable in size. She lived a normal life without any symptoms nor liver dysfunction.

## Discussion

The clinical presentation of IHCC is non-specific and insufficient to establish a definitive diagnosis. It is usually asymptomatic until late in course of disease and patients mostly presented with locally advanced tumors or large unresectable tumor resulting in poor disease prognosis. IHCC originates from the bile duct epithelium, and it sometimes forms a large mass in the liver like this case, who had less symptoms and almost normal hepatic functions even though tumor diameter was 15 cm in the liver hilum. The difficulty of early detection may worsen the prognosis of IHCC and made it an inoperable case to be treated by systemic chemotherapy. GC (Gemcitabine and Cisplatin) therapy was introduced with better survival effects, *i.e.* MST was 13.6 months.^1^ Recently, study of GCS (GS+S-1) therapy demonstrated the superiority over GS in the overall survival.^2^ However, this patient was deemed refractory to chemotherapy after five cycles of GC.

Radioembolization therapy has been actively carried out in treatment of IHCC with good survival outcomes (MST 10.3–22 months).^3,4^ However, high doses is required and adverse events cannot be ignored.^4^ As to the TACE, there are some reports^5–8^ showed survival merits and also suggested as one of the locoregional therapy in EASL (European Association for the study of the liver) guideline on IHCC.

One of the disadvantages of TACE using Lipiodol and gelatin sponge particle is irreversible damage of liver functions by repetition of treatment. In this case report, TACE procedures were all done by permanent drug-eluting spherical embolic material, which delivers high concentration of chemotherapeutic agents in the tumor and does not go into the sinusoid or peribiliary arterial plexus. The damage to normal liver parenchyma is well demonstrated in the liver functions test and no adverse event reported, while the tumor size decreased from 15 to 10 cm with complete necrosis. The patient still survives during the write-up of this report and from the first TACE treatment, she lives longer than 4 years in good condition. Previous reports from Aliberti et al^9^ and Poggi et al,^10^ the median survival treated by the same spherical embolic material were 13 months and 30 months, respectively. These studies concluded superiority of TACE using spherical embolic material over systemic chemotherapy.^9,10^ However, it is difficult to compare due to the difference of patients’ background. The best treatment method of TACE must be further investigated.

The antineoplastic agents used were doxorubicin and 5-FU, which have been used for the treatment of gastrointestinal tumors for a long time. The dose of each drug was chosen according to that of hepatocellular carcinoma treatment. By repeating this treatment, tumor shrinkage and necrosis were obtained. The reason for choosing this drug was that GC therapy did not respond in previous treatments. The dose was less than half that of systemic chemotherapy, and the side-effects of this treatment were mild and did not require any treatment. Drawback of this treatment was to repeat the treatment every few months. In order to compensate for this disadvantage, an inhibitor of angiogenesis, bevacizumab was combined with antineoplastic agents for transarterial infusion. After this attempt, all lesions in the liver had been well-controlled for one-and-half year until local recurrence. But, it was controlled by embolization with same drugs and embolic material, with additional embolization to the right inferior phrenic artery. 8 months after the final treatment, there are no signs of recurrence and no increase in small intrahepatic metastases. No liver dysfunction occurred during 4 years treatment period (Table 1).

In cases of advanced IHCC, TACE with spherical embolic material is preferable to avoid liver damage and to repeat the procedure especially with intra-arterial administration of VEGF inhibitor to prolong treatment interval.

## Conclusion

Even though complete remission cannot be expected in cases of IHCC, TACE using spherical embolic material may provide an effective local disease control with good quality of life and safety profile for unresectable IHCC patients refractory to systemic chemotherapy. Large and appropriate studies are required to provide evidence for making it a standard of care guidelines.

## Learning point

Chemoembolization with HepaSphere is safe and effective for local tumor control and demonstrated a good survival.
